# Molecular signatures and lineage diversification of neurogenic and gliogenic radial glia in the gyrencephalic ferret cortex

**DOI:** 10.21203/rs.3.rs-8062841/v1

**Published:** 2025-11-28

**Authors:** Jialin Li, Feihong Yang, Weiwei Li, Tongye Fu, Zhenmeiyu Li, Zizhuo Sha, Wenhui Zheng, Chuannan Yang, Jingzhe Yu, Danyu Han, Xin Jiang, Yan You, Xiaosu Li, Tong Ma, Miao He, Zhejun Xu, Xiaolei Song, Bin Chen, Jing Ding, Xin Wang, Zhuangzhi Zhang, Zhengang Yang

**Affiliations:** 1 Department of Neurology, State Key Laboratory of Brain Function and Disorders, Ministry of Education Frontiers Center for Brain Science, Institutes of Brain Science, Zhongshan Hospital, Fudan University, Shanghai 200032, China; 2 Department of Rehabilitation Medicine, Zhongshan Hospital, Fudan University, Shanghai 200032, China; 3 Institute of Pediatrics, National Children’s Medical Center, Children’s Hospital of Fudan University, Shanghai 201102, China; 4 Center for Clinical and Translational Medicine, Shanghai University of Medicine and Health Sciences, Shanghai 200237, China; 5 Department of Molecular, Cell and Developmental Biology, University of California Santa Cruz, Santa Cruz, CA 95064, USA.

**Keywords:** cortical development, cortical evolution, gyrencephalic, ferret, neurogenesis, gliogenesis, ERK, PKA, YAP, SHH signaling, outer radial glia

## Abstract

Human exceptional cognition stems from evolutionarily derived cortical adaptations that drive expansive neurogenesis. In this study, we employ the gyrencephalic ferret model to systematically characterize the molecular profiles and lineage dynamics of cortical radial glia (RGs). By applying scRNA-Seq to ferret and human cortices, we identify conserved regulatory programs underlying cortical neurogenesis and gliogenesis in mammals. Through integrated scRNA-Seq, BrdU labeling, and immunohistochemical approaches, we show that, similar to their human counterparts, ferret cortical outer radial glia (oRGs), exhibit enhanced ERK and PKA signaling. ERK and PKA act in a mutually reinforcing manner to boost oRG self-renewal and neurogenesis, while inhibiting gliogenesis and prolonging the neurogenic period. Furthermore, we identify regional specialization within cortical gliogenic RGs: YAP/TAZ activation drives ventricular zone truncated radial glia (tRGs) toward ependymal glial fate in medial cortex, whereas SHH signaling instructs lateral cortical tRGs to generate tripotential intermediate progenitor cells, which serve as a shared source of astrocytes, oligodendrocytes, and cortically-derived olfactory bulb interneurons. Our findings support a model in which mammalian cortical neurogenesis, gliogenesis, and evolutionary expansion are co-regulated through an integrated signaling network orchestrated by ERK, PKA, YAP/TAZ, and SHH. This network relies on a precisely balanced interplay of mutual inhibition among these pathways to ensure proper developmental outcomes.

## INTRODUCTION

In the developing telencephalon, radial glia (RGs) function as the primary neural stem cells, capable of self-renewal and multipotency^1–8^. In the mouse cortex, individual cortical RGs typically produce 8–9 glutamatergic pyramidal neurons (PyNs)^9^. Upon the completion of neurogenesis, a specified population of cortical neurogenic RGs undergoes gliogenic transition to generate cortical glial cells, including macroglia (astrocytes and oligodendrocytes)^9,10^ and ependymal glial cells^11–13^. Throughout cortical development, mouse cortical RGs maintain their characteristic full-span morphology, extending processes to both the ventricular and pial surfaces. Throughout cortical development, the majority of mammalian RGs undergo asymmetric division to generate PyNs and glial cells indirectly through the production of intermediate progenitor cells (IPCs)^1–6,14^.

In mouse, at mid-corticogenesis, the transition stages between neurogenesis and gliogenesis, three distinct types of RGs can be classified based on their cell lineage, which we recently designated as N-RGs, E-RGs, and T-RGs^15^. N-RGs generate PyN-IPCs, while E-RGs generate ependymal cells^12,13^, and T-RGs generate cortical tripotential IPCs (Tri-IPCs) that express *Ascl1, Egfr*, and *Olig2*^4,16–21^. Tri-IPCs give rise sequentially to astrocyte lineage-restricted IPCs (APCs), oligodendrocyte lineage-restricted IPCs (OPCs), and IPCs destined for generating cortically derived olfactory bulb interneurons (OBIN-IPCs). These lineage-restricted IPCs then divide symmetrically to generate cortical astrocytes, oligodendrocytes, and OBINs, respectively^16–23^.

During human corticogenesis, full-span radial glia (fRGs) persist from gestational week (GW) 8 until GW16, after which they gradually bifurcate to produce truncated radial glia (tRGs) in the ventricular zone (VZ) and outer radial glia (oRGs, or basal radial glia) in the outer subventricular zone (OSVZ)^21,24–29^. Human cortical oRGs are a key driver of cortical expansion. While scarce in mice, they are found abundantly in primates and are neurogenic, primarily generating PyN-IPCs that give rise to upper-layer pyramidal neurons^4,15,18,20,21,29^. Human cortical tRGs undergo a developmental switch to acquire gliogenic potential. Specifically, tRG subsets in the medial cortex adopt an E-tRG identity to produce ependymal cells^15,17,21,30,31^, whereas their lateral counterparts transition into a T-tRG fate, giving rise to Tri-IPCs^15,19,21^.

The evolutionary trajectories of mammalian species provide critical insights into the complex organization of the human cerebral cortex. In this study, we utilize the gyrencephalic ferret (*Mustela putorius furo*) model to systematically characterize the molecular signatures and lineage trajectories of cortical RGs. Our cross-species comparative analyses reveal conserved mechanisms underlying cortical neurogenesis, gliogenesis, and evolutionary expansion between ferrets and humans, highlighting fundamental principles of mammalian cortical development.

## RESULTS

### scRNA-seq reveals molecular signatures of ferret cortical oRGs, E-tRGs, and T-tRGs

**Figure 1.**
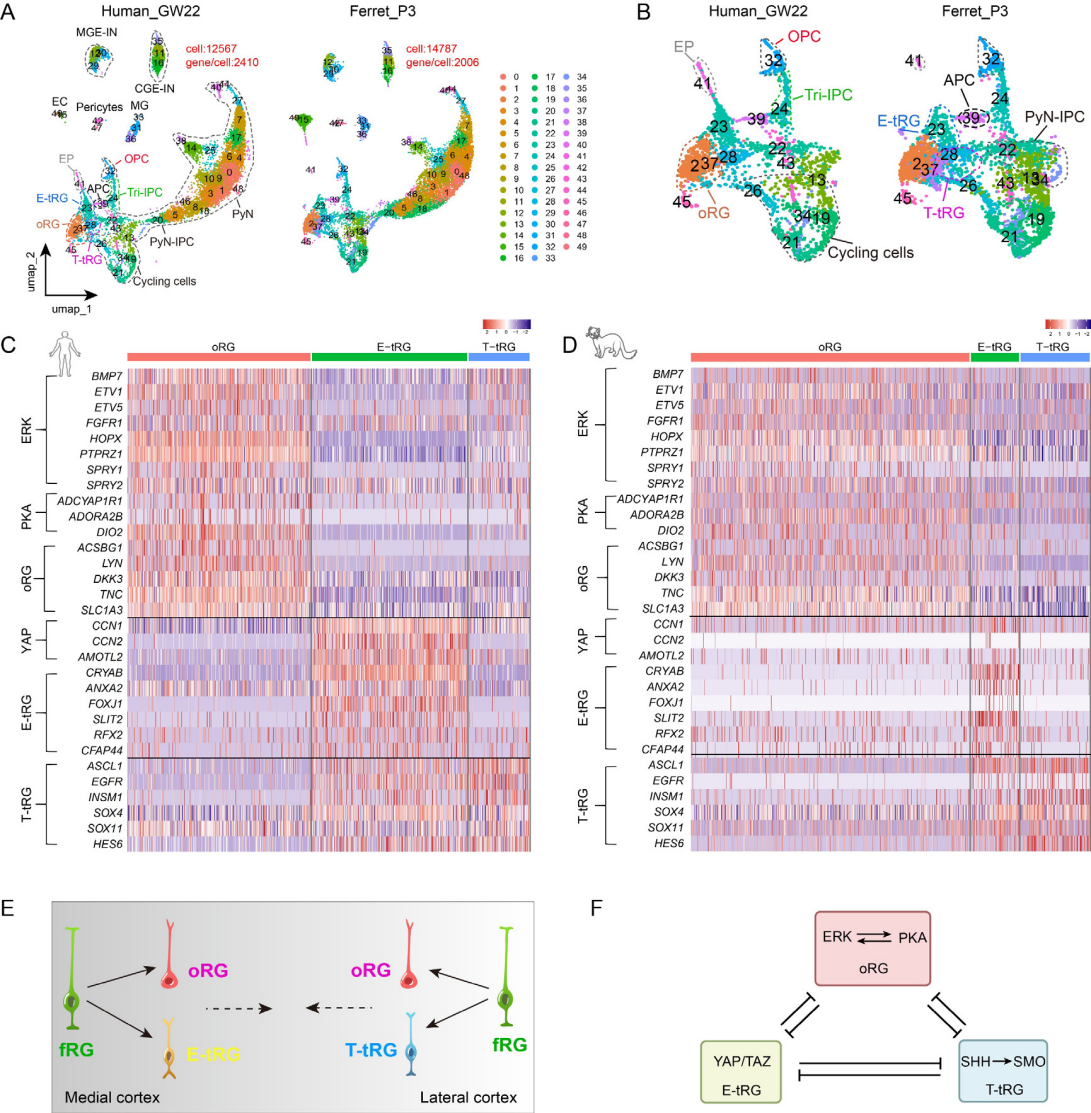
The molecular signatures of human and ferret cortical oRGs, E-tRGs, and T-tRGs. (**A**) UMAP projection of cross-species scRNA-seq data, colored by cell cluster identity. CGE-IN, caudal ganglionic eminence interneuron; MGE-IN, medial ganglionic eminence interneuron; EC, endothelial cell; EP, ependymal cells; MG, microglia. (**B**) Enlarged region of (**A**) displaying RG and IPC subpopulations. (**C, D**) Heatmap showing conserved gene expression patterns across homologous cortical populations (oRGs, E-tRGs, T-tRGs) in human (GW22) and ferret (P3) cortices. (**E**) Model of cortical RG differentiation hierarchy showing fRGs as progenitors generating oRGs, E-tRGs, and T-tRGs populations during human and ferret corticogenesis. (**F)** Cortical oRGs maintain elevated ERK and PKA pathway activity, whereas E-tRGs display enhanced YAP signaling. T-tRGs progressively activate SHH signaling. These distinct cellular states are stabilized through mutual inhibition among these signaling pathways.

As a gyrencephalic species, the ferret is a suitable model for studying cortical development and evolution, possessing fRGs, oRGs and tRGs that closely resemble their human counterparts^32–36^. However, recent scRNA-seq studies of developing ferret cortex have failed to precisely define the molecular identity of cortical oRGs^30,37^. To address this gap, we conducted high-resolution scRNA-seq analysis of postnatal day 3 (P3) ferret cerebral cortex, which developmentally corresponds to human cortex at GW22^38^. After filtering out low-quality/doublet cells, a total of 14,787 cells were recovered with a median of 2,000 transcribed genes per cell (Fig. 1A, B). By integrating single-cell transcriptomic data from developing P3 ferret cortex (newly generated in this study) and GW22 human cortex (published data)^39^, we identified evolutionarily conserved molecular signatures of cortical oRGs, E-tRGs, T-tRGs, Tri-IPCs, APCs, and OPCs across species (Fig. 1, Fig. S1–S3, Table S1–S3).

Ferret cortical oRGs at P3 maintain high extracellular signal-regulated kinase (ERK) signaling and protein kinase A (PKA) signaling, which consequently suppresses Sonic Hedgehog (SHH) signaling and YAP signaling (YAP/TAZ activity)^15^. First, ferret oRGs exhibit elevated expression of ERK signaling-responsive genes (*BMP7, ETV1, ETV5, HOPX, PTPRZ1*, and *SPRY1/2*) compared to E-tRGs and T-tRGs (Fig. 1A–D, Fig. S1)^15,20,29^. Second, oRGs demonstrate enhanced cAMP-PKA signaling activity, evidenced by higher expression of *ADCYAP1R1, ADORA2B*, and *DIO2* (Fig. 1D, Fig. S1). cAMP-PKA signaling could be activated through ADCYAP1R1 binding to PyN-derived ADCYAP1, which triggers GNAS (Gsα)-mediated stimulation of adenylyl cyclase activity^40,41^. Likewise, adenosine-ADORA2B binding also activates cAMP-PKA signaling^42,43^. The *DIO2* promoter contains a conserved canonical cAMP response element across humans, rats, and mice^44–46^, making it a reliable readout of cortical cAMP-PKA-CREB signaling activity^44–46^. Third, classical studies have established that ERK and PKA signaling pathways can engage in mutual reinforcement^47–49^. Fourth, the PKA pathway exerts robust inhibitory control over SHH-SMO signal transduction^50–54^, and PKA is additionally established as a potent inhibitor of YAP signaling^52,55–57^. Thus, elevated ERK and PKA activity constitutes a hallmark characteristic of ferret cortical oRGs. Furthermore, compared to E-tRGs and T-tRGs, ferret cortical oRGs also expressed higher levels of *ACSBG1, LYN, DKK3, TNC*, and *SLC1A3* among others; these genes are also highly expressed by human cortical oRGs^29,58^. Taken together, our analysis revealed evolutionarily conserved molecular signatures between ferret and human cortical oRGs (Fig. 1, Fig. S1, Table S1, S2).

Ferret cortical E-tRGs exhibit elevated YAP signaling, as they expressed higher levels of YAP target genes, including *CCN1* (*CYR61*), *CCN2* (*CTGF*), and *AMOTL2* Fig. 1D), compared with oRGs and T-tRGs. MST1/2-LATS1/2-mediated YAP/TAZ phosphorylation restricts nuclear entry, while nuclear YAP/TAZ-TEAD complexes activate proliferative and homeostatic programs^52,55–57,59^. Accordingly, YAP signaling activation promotes expression of E-tRG and early ependymal cell markers, including *CRYAB, FOXJ1, ANXA2, SLIT2, RFX2*, and *CFAP44* (Fig. 1D, Fig. S1). These observations are consistent with the previous result that YAP signaling is a critical driver of cortical ependymal cell production^15,60,61^. On the other hand, E-tRGs exhibit markedly reduced PKA and ERK signaling activity (Fig. 1C, D), consistent with established reciprocal inhibition between these pathways. Previous work has shown that PKA activation downregulates YAP signaling^52,55–57,59^, while conversely, YAP activation represses both cAMP-PKA and ERK pathways^62,63^. Furthermore, YAP activation has been shown to disrupt primary cilium formation^64^, potentially attenuating SHH-SMO pathway activity. Together, these findings establish YAP signaling as a defining characteristic of E-tRGs, mediated through its inhibitory effects on PKA/ERK and SHH signaling cascades.

Both loss- and gain-of-function studies reveal that SHH signaling primarily upregulates *Ascl1* and *Egfr* expression in mouse cortical progenitors during the neurogenesis-to-gliogenesis transition—a finding consistent with the expression of these markers in ferret primed cortical T-tRGs at P3 (Fig. 1D, S1)^15,16,18,20,21,29,65,66^. Ferret T-tRGs additionally initiate expression of *INSM1, SOX4, SOX11*, and *HES6*, key transcriptional regulators that promote the transition from quiescent stem cell states to primed states and facilitate IPC generation (Fig. 1D). During cortical development, the mouse brain demonstrates progressively elevated SHH signaling activity^67^. This pattern is evolutionarily conserved, as human cerebral cortex development shows similar characteristics^15^. We propose that ferrets likewise exhibit this progressive SHH signaling upregulation during their cortical development. Elevated SHH-SMO signaling in ferret cortical T-tRGs represses PKA activity (downregulation of *DIO2*) through an evolutionarily conserved reciprocal inhibition mechanism^50,51,54,59^. PKA downregulation also leads to concomitant reduction in ERK signaling^47–49^. Experimental evidence shows bidirectional suppression between YAP and SHH during cell type specification^68^. As demonstrated in cortical studies, YAP drives E-tRG-derived multiciliated ependymal lineages^15,60,61^, while SHH-SMO promotes primary-ciliated T-tRGs generation^15,16,18,20,29^.

Above comparative analyses demonstrate that human and ferret cortical RG subtypes share conserved molecular signatures, however, specific markers, including ERK/PKA signaling components and their downstream targets (*BMP7* and *DIO2*) (Fig. 1C, D, Table S3), exhibit significantly elevated expression in human cortical oRGs compared to ferret oRGs^15,20,29^. Furthermore, we identified a subset of genes expressed in human cortical RGs but absent in their ferret counterparts (Fig. S2A, B). For example, *CXCL14* is specifically expressed in human oRGs, while *CXCL12* marks human E-tRGs, neither is detected in ferret RGs. *PDGFD* is present in multiple human cortical RG subtypes but undetectable in ferret RGs^69,70^. Additional genes *CD9, CRH, FAM107A, GPX3, LRRC17*, etc.) show high expression in human oRGs but are minimally or not expressed in ferret oRGs (Fig. S2, Table S3). Notably, many of these human-specific RG genes are functionally linked to key signaling pathways, including PKA, ERK, YAP, and SHH. These differential gene expression patterns between human and ferret RGs highlight how evolutionary innovation in cortical development emerges not from new genes, but rather through the strategic rewiring and optimization of existing gene regulatory network combinations within neural progenitor populations.

In the human cortex, cortical fRGs (full-span radial glia) are depleted following their generation of oRGs and tRGs^21,26^. In contrast, ferret cortical development shows distinct persistence of fRGs: while a substantial population of fRGs generate oRGs and tRGs, a significant fRG fraction remains detectable even through at least P8^30,37,70,71^. We hypothesize that the remaining fRGs in the later-stage ferret cortex exhibit molecular and functional similarities to neurogenic oRGs. This phenotypic overlap likely obscured their detection in our scRNA-seq analysis, preventing clear identification of fRG-specific signatures.

In summary, we conclude that the molecular identity of oRGs, E-tRGs, and T-tRGs is largely conserved between humans and ferrets, with species differences restricted to a subset of genes (Fig. 1E, F). Notably, oRGs consistently exhibit elevated ERK/PKA signaling activity, E-tRGs are molecularly defined by predominant YAP signaling, while T-tRGs demonstrate enhanced SHH pathway activation (Fig. 1F)^15^. These conserved signaling signatures suggest deep evolutionary preservation of core regulatory mechanisms, while species-specific genetic variations likely contribute to divergent cortical developmental outcomes.

### Molecular identities of ferret cortical Tri-IPCs, APCs, and OPCs

Cortical oRGs exhibit neurogenic capacity, generating cortical PyNs but not glia or GABAergic interneurons^4,15,18,20,21,29^, while E-tRGs specifically produce ependymal cells^15^. Consistent evidence has shown that primed cortical T-tRGs expressing *ASCL1* and *EGFR* generate Tri-IPCs that maintain *ASCL1/EGFR* expression while additionally acquiring *OLIG1/2* expression^4,15,17–19,21,29^. scRNA-seq profiling revealed striking molecular conservation between human and ferret cortical Tri-IPCs (Fig. S3). Tri-IPCs in both species showed characteristic high expression of core regulators (*ASCL1, EGFR, OLIG1/2*) and shared elevated expression of additional markers including *GAS1, DLL1, DLL3*, among others (Fig. S3A-D). Previous studies show that, in both mouse and human cortices, Tri-IPCs subsequently generate three distinct types of progeny: APCs, OPCs, and OBIN-IPCs^4,16–19,21,29,39^. Our scRNA-seq analysis of ferret P3 cortex also provides strong evidence that Tri-IPCs generate both APCs and OPCs (Fig. S3A-D). The *ASCL1*^+^*EGFR*^+^*OLIG1/2*^+^ Tri-IPCs differentiate into APCs that exhibit progressive downregulation of *ASCL1/EGFR/OLIG1/2* and progressive upregulation of astrocyte markers *HES1, FGFR3, ITGA6*, and *GFAP* Fig. S3). Tri-IPCs also differentiate into OPCs maintaining *ASCL1* and *OLIG1/2* expression while acquiring canonical oligodendrocyte lineage markers (*PDGFRA, APOD, PCDH15, SOX10, NKX2–2*) (Fig. S3). Notably, our P3 scRNA-seq datasets did not capture OBIN-IPC clusters derived from Tri-IPCs (Fig. S3), suggesting these lineages are generated sequentially (first APCs, then OPCs, followed by OBIN-IPCs)^4,18^. This temporal pattern was confirmed by BrdU labeling at P8, which clearly identified newly generated OBIN-IPCs in the ferret cortical SVZ (see below). In conclusion, our comparative scRNA-seq analysis of ferret and human cortices demonstrates striking evolutionary conservation in both the molecular signatures and lineage trajectories of Tri-IPCs and their progeny, APCs, OPCs, and potentially OBIN-IPCs (Fig. S3).

### In vivo identification of oRGs and their neuronal lineage progression in ferret cortical development

**Figure 2.**
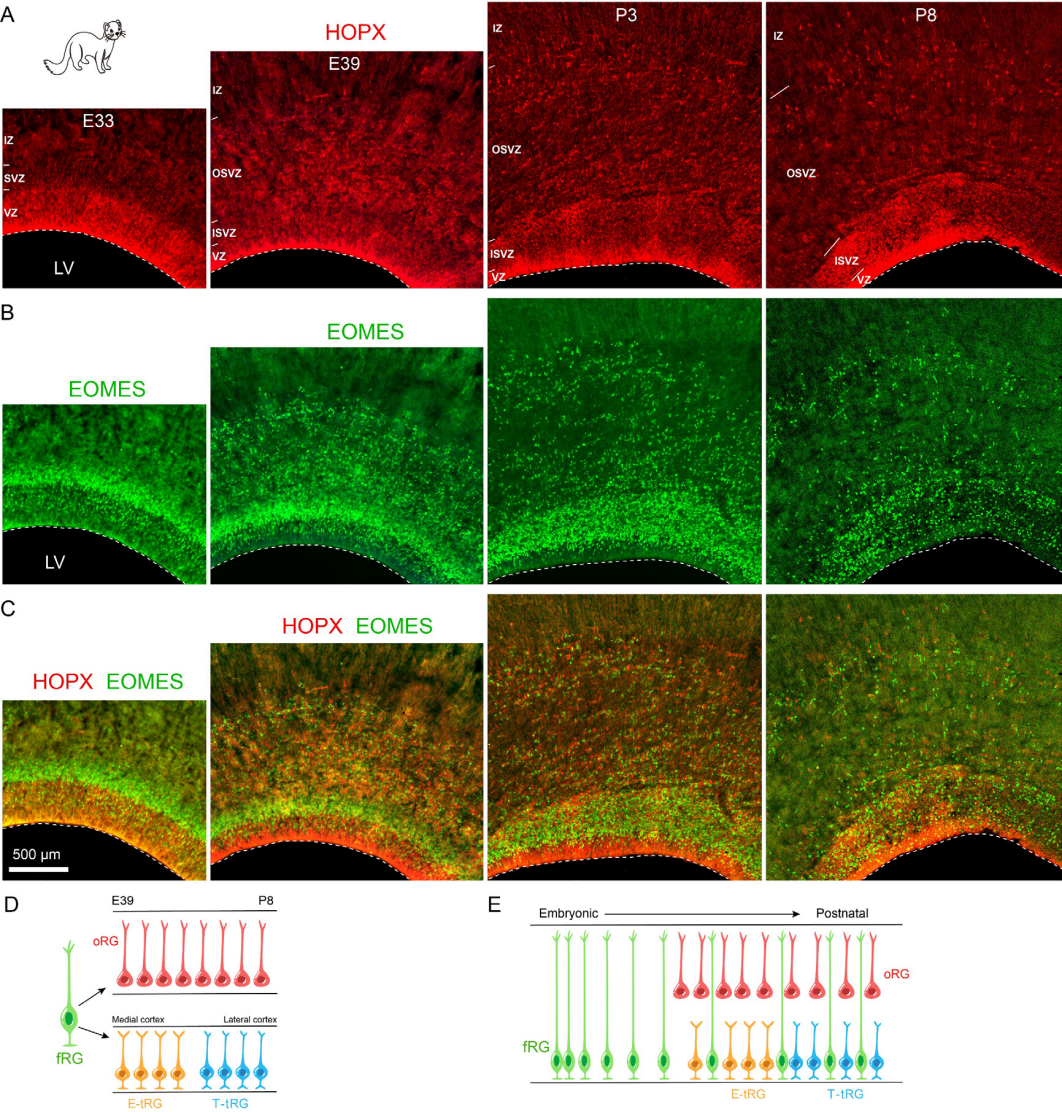
Identification of oRGs in developing ferret cortex. **(A-C)** HOPX and EOMES (TBR2) double immunostaining demonstrates the exclusive presence of fRGs in the E33 ferret cortex. **(D)** Schematic representation of cortical oRGs maintenance from E39 through P8, as evidenced by above immunohistochemical analysis. (**E**) According to published studies, a subpopulation of ferret cortical fRGs (full-span RGs) persists from E33 through P8. Thus, during late ferret cortical development, fRGs, oRGs, E-tRGs, and T-tRGs co-localize in the cortex. IZ, intermediate zones; LV, lateral ventricle.

To confirm the ferret oRG molecular profiles derived from scRNA-seq data, we examined the expression of established oRG marker proteins via immunofluorescence staining of ferret cortical tissue at E33 (n = 4), E39 (n = 3), P3 (n =2) and P8 (n = 2) (Fig. 2A). Mammalian cortical oRG strongly express HOPX, making it an excellent marker for identifying oRG populations, especially when combined with their anatomical positioning^21,58^. At E33, HOPX-expressing cells are primarily restricted to the VZ of the ferret cortex, demonstrating that cortical fRGs express HOPX while oRGs have not yet emerged at this developmental stage (Fig. 2A). EOMES (TBR2)-positive cells are predominantly localized in the subventricular zone (SVZ), consistent with the absence of oRGs at E33 (Fig. 2A–C). We further analyzed the expression patterns of HOPX and EOMES at E39, P3, and P8. From E39 through P8, we observed HOPX+ cells in the VZ and EOMES+ cells in the SVZ, as well as both HOPX+ and EOMES+ cell populations in the OSVZ (Fig. 2A). These findings provide strong evidence that oRGs persist in the ferret cortical OSVZ for at least a 12-day period spanning late embryonic to early postnatal development (Fig. 2B). Triple-immunostaining on P3 ferret cortical sections confirmed that HOPX+ oRGs express PAX6 but lack EGFR expression (Fig. S4A-D). Notably, these oRGs exhibited positive staining for pERK (phosphorylated ERK) (Fig. S4E), indicating elevated ERK signaling activity. These findings are consistent with our scRNA-seq data analysis.

**Figure 3.**
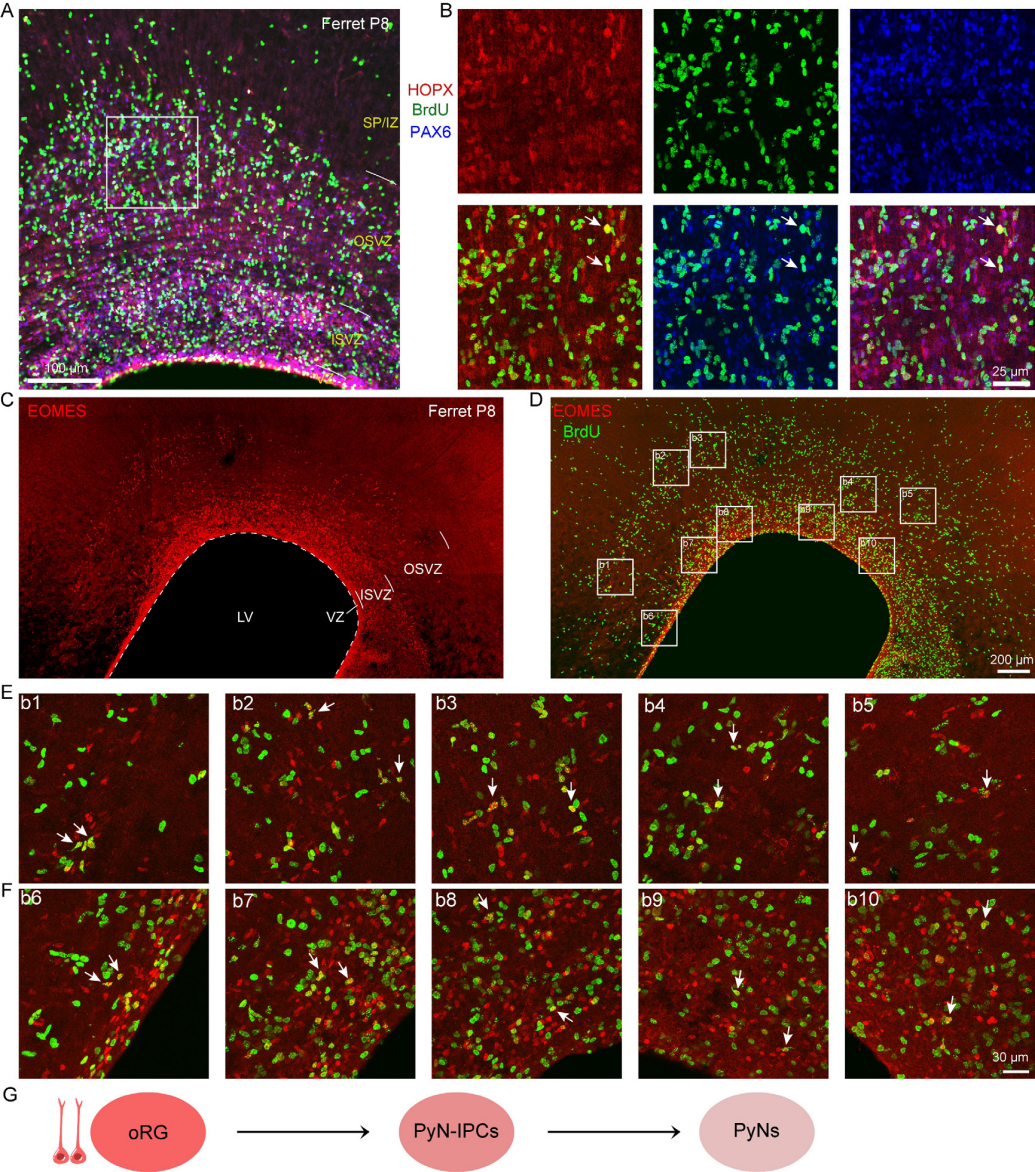
Self-renewal and neurogenic capacity of cortical oRGs in postnatal ferrets. **(A, B)** Triple immunostaining for BrdU/HOPX/PAX6 in P8 ferret cortex 24 hours after BrdU injection (P7), revealing proliferating oRGs (BrdU+/HOPX+/PAX6+ cells, arrows) in the OSVZ. **(C-F)** BrdU/EOMES co-staining demonstrates BrdU-incorporated cells (BrdU+/EOMES+ cells, arrows) across both OSVZ and SVZ at P8. **(E)** Schematic model of oRG lineage progression.

To evaluate proliferative activity, we conducted BrdU labeling at P7 and analyzed the cortices 24 hours later (P8). Immunostaining revealed BrdU/HOPX/PAX6 triple-positive cells in the OSVZ (Fig. 3A, B), confirming that a subset of oRGs retains proliferative capacity, indicative of sustained self-renewal potential. We also observed BrdU/EOMES double-positive cells in the OSVZ, likely representing newly generated PyN-IPCs derived from asymmetrically dividing oRGs (Fig. 3C–E). These results provide direct evidence that ferret cortical oRGs preserve neurogenic competence at P8, continuing their production of PyNs (Fig. 3G).

**Figure 4.**
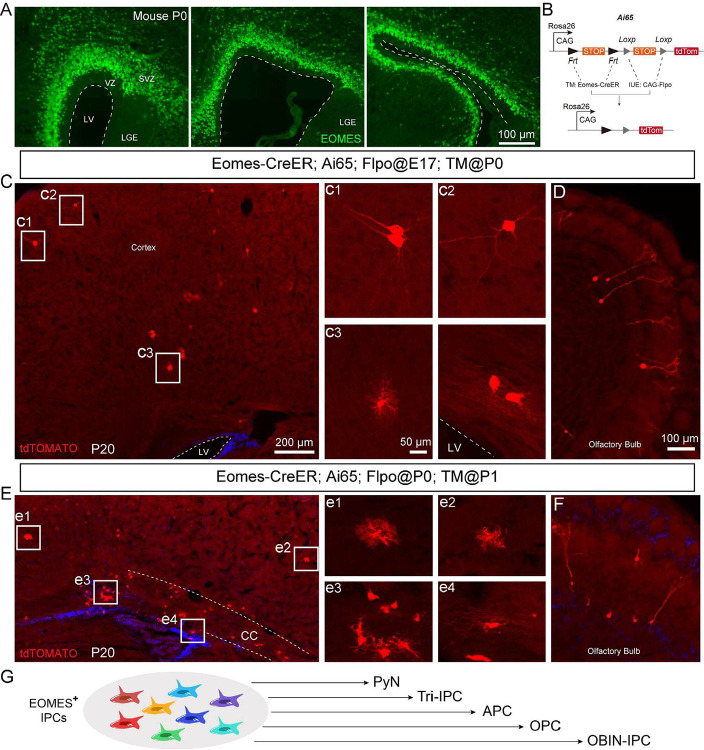
Lineage potential of EOMES+ cells in neonatal mouse cortical SVZ. **(A)** EOMES+ cells in P0 mouse cortical SVZ. **(B)** Genetic strategy for labeling EOMES+ cells (*Eomes-CreER; Ai65* mice). **(C-F)** Fate mapping via IUE (E17/P0) of *pCAG-Plpo* plasmids followed by tamoxifen induction (P0/P1). By P20, tdT+ progeny included: upper-layer cortical PyNs (c1, c2), cortical astrocytes (c3, e1, e2), OPCs and oligodendrocytes in the corpus callosum (e3, e4), along with OBINs (**D, F**). (**G**) Developmental potential of EOMES+ progenitors in the neonatal mouse cortical SVZ. CC, corpus callosum; LGE, lateral ganglionic eminence.

Interestingly, we also detected BrdU/EOMES double-positive cells in the inner subventricular zone (ISVZ) of P8 ferret cortex (Fig. 3F). We propose that at this late developmental stage, EOMES+ cells in the ferret ISVZ may represent a distinct progenitor population, rather than serving as a marker for PyN-IPCs as observed during earlier developmental periods. To test this hypothesis, we employed a mouse genetic model to specifically label EOMES+ cells in P0 and P1 cortex, and performed lineage tracing to determine the ultimate fate of these EOMES-expressing progenitors (Fig. 4A, B). We used *Eomes-CreER; Ai65* double transgenic mice for these lineage tracing experiments^72^. The *Ai65* reporter mouse line contains two independent transcriptional stop cassettes: an FRT-flanked STOP sequence and a loxP-flanked STOP sequence, which jointly suppress tdTomato (tdT) expression. Upon FLP- and Cre-mediated recombination, excision of both STOP cassettes permits tdT expression exclusively in cells that actively express both recombinases (Fig. 4B). To specifically label dorsal cortical RG-derived EOMES+ cells, we performed in utero electroporation (IUE) at E17.0 or P0 to deliver *pCAG-Plpo* plasmids into the cortical VZ of *Eomes-CreER; Ai65* embryos. Tamoxifen was subsequently administered via intraperitoneal (i.p.) injection at P0 or P1, respectively, to induce CreER activity (Fig. 4B). In this experimental paradigm, a subset of EOMES+ cells at P0/P1 derived from electroporated cortical RGs exhibited tdT expression. By P20, we identified multiple tdT+ lineages: PyNs (predominantly in upper cortical layers), astrocytes, OPCs, and mature oligodendrocytes in the cortex or in the corpus callosum, along with OBINs in the OB (Fig. 4C–F). These lineage tracing experiments demonstrate that EOMES+ cells in the mouse postnatal cortical SVZ do not exclusively represent PyN-IPCs. Rather, they constitute a heterogeneous progenitor pool derived from late-stage cortical RGs, comprising multiple lineage-committed progenitors including PyN-IPCs, Tri-IPCs, APCs, OPCs, and OBIN-IPCs (Fig. 4G). This developmental phenomena may extend to human and ferret cortical development. Specifically, the BrdU/EOMES double-positive cell population identified in the P8 ferret SVZ similarly exhibits heterogeneous progenitor characteristics rather than displays a predominant PyN-IPC identity.

### Identification of E-tRGs and their differentiation trajectory toward ependymal lineages in the developing ferret cortex

**Figure 5.**
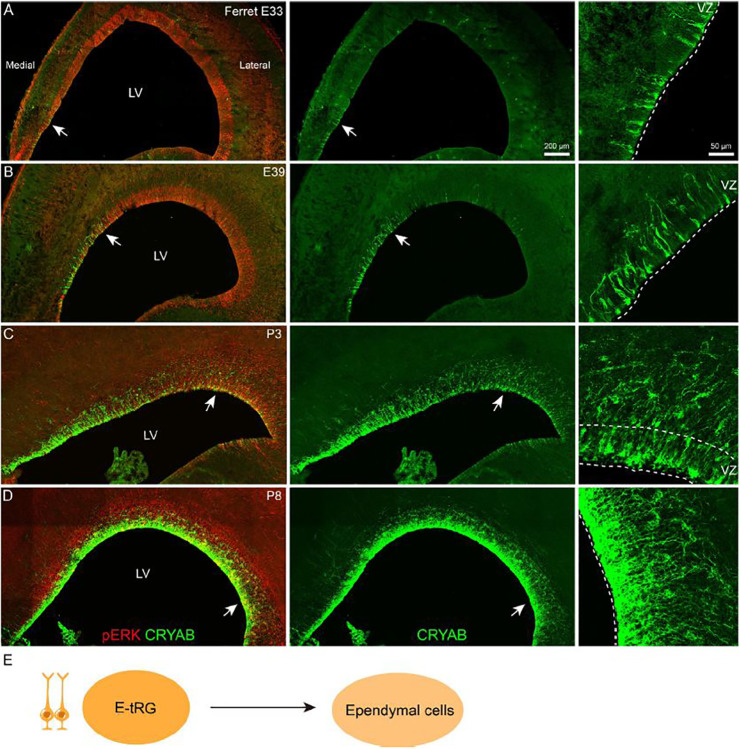
Spatiotemporal expansion of CRYAB+ E-tRGs across developing ferret cortex. **(A-D)** CRYAB/pERK co-immunostaining analysis from E33 to P8 reveals complementary expression patterns, with CRYAB showing medial-high distribution and pERK exhibiting lateral-high gradient. **(E)** Lineage specification pathways of cortical E-tRGs.

In the mouse brain, ependymal cell development surrounding the lateral ventricle generally progresses in three spatial gradients: medial-to-lateral, caudal-to-rostral, and ventral-to-dorsal^31^. YAP signaling is essential for cortical E-RG specification and ependymal cell differentiation^15,60,61^, mediated at least partially through induction of key transcription factors (i.e., *Foxj1*) and other ependymal markers including *Cryab*, *S100a11*, and *Ogn*^15^. Human cortical scRNA-seq data identify *CRYAB* as an early marker of E-tRGs and immature ependymal cells, while *FOXJ1* exhibits temporally delayed yet overlapping expression in the same lineage^15,21,26^. *CRYAB* additionally identifies a subpopulation of T-tRGs, while *FOXJ1* serves as a persistent marker for mature ependymal cells^15,21,26,30,31,37^. We therefore employed immunostaining of CRYAB and FOXJ1 to examine both the spatial distribution and temporal progression of ependymal cell development in ferret and human cortex (Fig. 5, Fig. S5). At E33, sparse CRYAB+ cells were detected in the ferret medial cortex (Fig. 5A). From E39 to P3, CRYAB+ cells progressively expanded from medial to lateral regions along the cortical ventricular surface (Fig. 5B, C). By P8, CRYAB expression became widespread, marking the entire population of cortical E-tRGs and a subset of T-tRGs (Fig. 5D). FOXJ1 displayed a similar spatial expression gradient but with markedly reduced intensity (Fig. S5A-D). Notably, even at P8, FOXJ1 expression remained restricted to medial E-tRGs (Fig. S5D). These results indicate that YAP signaling also exhibits a medial-strong to lateral-weak gradient in the developing cortical VZ, as YAP is required for ependymal cell development^15,60,61^. YAP signaling directly or indirectly suppresses PKA and ERK signaling pathways^62,63^. Consistent with this regulatory relationship, pERK immunostaining reveals a complementary mediolateral gradient in the developing ferret cortical VZ, showing lowest activity medially and highest laterally, which inversely correlates with the YAP signaling gradient (Fig. 5, Fig. S5). Notably, this pERK staining pattern is evolutionarily conserved, as evidenced by an identical mediolateral gradient in the mouse cortical VZ (Fig. S6).

In the developing human cortex, our analysis suggests that sparse CRYAB+ fRGs can first be detected in the medial cortex at GW14 based on scRNA-seq data (Fig. S7)^73^. By GW19, CRYAB expression expands to nearly all cortical E-tRGs and a subset of T-tRGs, but not the ganglionic eminence regions (Fig. S7A). This pattern progresses further by GW23, when CRYAB becomes ubiquitously expressed throughout the VZ of the lateral ventricle, including the ganglionic eminence (Fig. S7C). This observation is further supported by bulk and scRNA-Seq analyses of the human fetal striatum^74^. Notably, FOXJ1 expression in the human cortical VZ lags significantly behind CRYAB. FOXJ1-immunoreactive cells were not detected in cortical RGs at GW19 and remained restricted to the medial cortical VZ even at GW23 (Fig. S7B, C). Furthermore, consistent with observations in mouse and ferret development, ERK signaling in the human cortex undergoes progressive downregulation in the cortical VZ (Fig. S8). At GW19, the majority of cortical tRGs exhibited pERK expression, albeit at reduced levels (Fig. S8A, B). By GW23, only a small subset of tRGs retained pERK expression (Fig. S8C-E). In contrast, oRGs in the OSVZ maintained robust pERK expression from GW19 through GW23 (Fig. S8B, D). Collectively, these findings demonstrate that mammalian cortical RGs generate ependymal cells in a conserved medial-to-lateral gradient, which is promoted by YAP signaling^15,60,61^.

### Identification of T-tRGs and their developmental trajectory toward Tri-IPCs in the ferret cortex

The emergence of EGFR+ cells in the mammalian cortical VZ and SVZ serves as a key hallmark, indicating that cortical RGs are primed to initiate the production of Tri-IPCs and subsequently generate APCs, OPCs, and OBIN-IPCs^19,21,75^. EGFR is also expressed in the substantial RGs and IPCs in the embryonic ganglionic eminence (Fig. S9A). While OLIG2+ OPCs and DLX2+ interneuron-IPCs from the ferret ganglionic eminence migrate into the cortex and maintain their OLIG2 or DLX2 expression, EGFR+ IPCs derived from the ganglionic eminence completely downregulate EGFR upon entering the cortex (Fig. S9A-D). Therefore, cortical EGFR+ cells must originate from cortical RGs. Furthermore, the expression gradient of EGFR+ cells in the P3 ferret cortical ISVZ shows higher density and greater numbers in the lateral SVZ compared to the medial cortex (Fig. S10A). This pattern suggests that ferret T-tRGs in the lateral cortex generate Tri-IPCs before those in the medial cortex (Fig. S10B), which contrasts with the ependymal cell production that proceeds from medial to lateral cortex (Fig. 5, Fig. S5).

**Figure 6.**
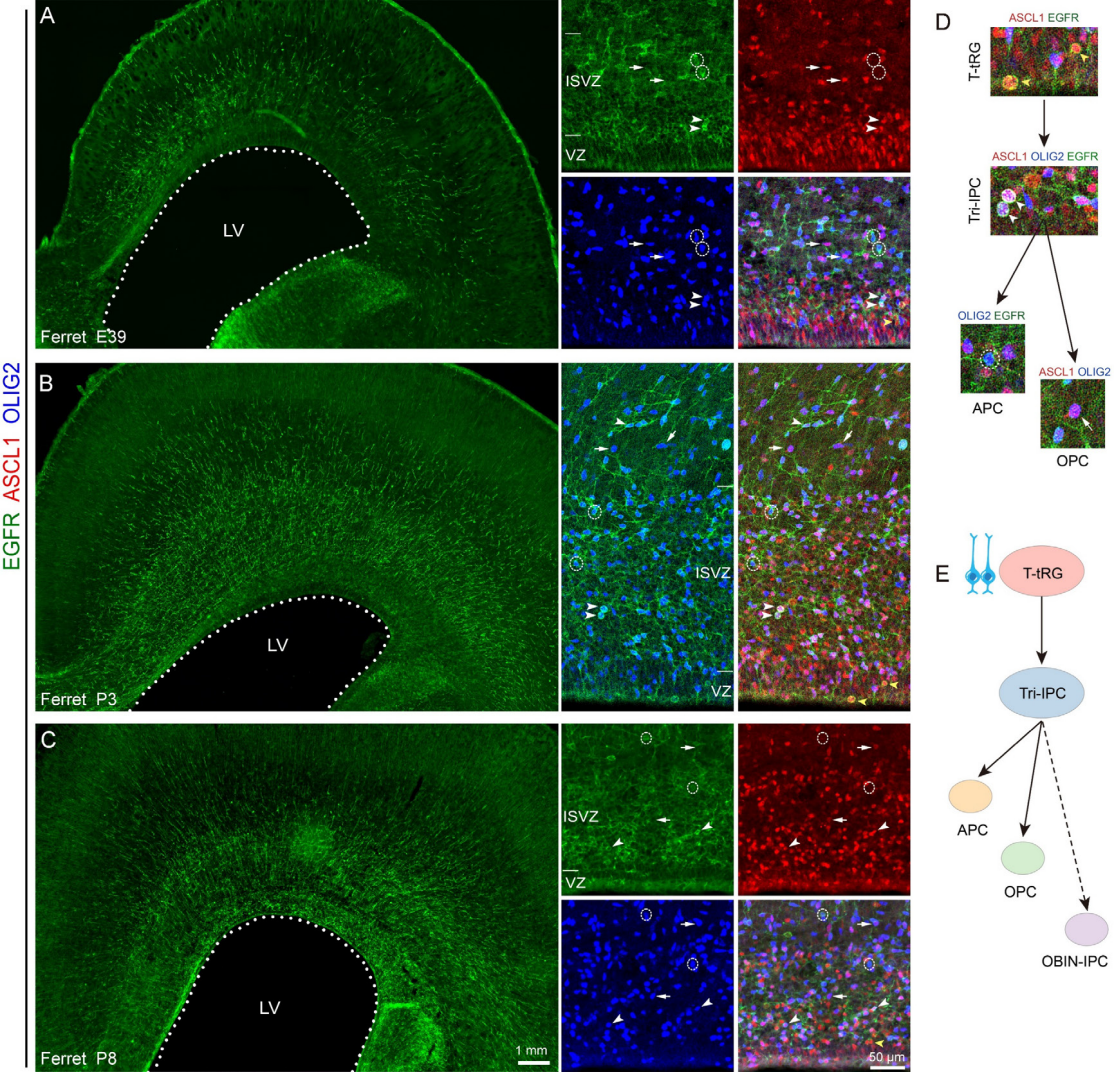
Characterization of T-tRGs and their lineage progression in developing ferret cortex. **(A-C)** Triple immunostaining for EGFR/ASCL1/OLIG2 identifies distinct progenitor populations across developmental stages (E39, P3, P8): EGFR+/ASCL1+ T-tRGs (color-coded arrowhead), EGFR+/ASCL1+/OLIG2+ Tri-IPCs (arrowheads), EGFR+/OLIG2+ APCs (circled cells), and ASCL1+/OLIG2+ OPCs (arrows). **(D)** Lineage hierarchy of EGFR+/ASCL1+ T-tRGs (color-coded arrowhead) with developmental relationships (all immunolabeled cells from P3 cortex in panel **B**). Note mitotic Tri-IPCs exhibiting large, spherical nuclei (arrowheads). **(E)** Proposed T-tRG lineage model integrating scRNA-seq and immunohistochemical data. In the P3 ferret cortex, Tri-IPCs have not yet begun large-scale production of OBIN-IPCs.

EGFR+ cells were undetectable in the ferret E33 cortex (Fig. S9A), consistent with the phase of cortical PyN genesis. At E39, oRGs first appeared in the OSVZ, concomitant with sparse EGFR+ cell distribution across the VZ (displaying weak immunoreactivity), ISVZ, and OSVZ (Fig. 6A). By P3–P8 stages, EGFR+ cells formed dense populations extending continuously from the ISVZ through OSVZ, (Fig. 6B–C). This distinct spatiotemporal progression precisely delineates the gliogenic peak during ferret cortical development. Throughout all developmental stages examined (E39, P3, P8) in ferrets, primed T-tRGs in the cortical VZ consistently exhibited relatively weak co-expression of ASCL1 and EGFR. As these EGFR+/ASCL1+ T-tRGs generated Tri-IPCs, they showed marked upregulation of ASCL1, EGFR, and OLIG1/2 expression (Fig. 6A–D). Notably, a subset of these Tri-IPCs displayed large, rounded nuclei and appeared as closely paired daughter cells, morphological features characteristic of cells completing symmetric division with high DNA content (Fig. 6D). These Tri-IPCs subsequently differentiated into: (1) EGFR+/OLIG2+ APCs with concomitant ASCL1 downregulation, and (2) ASCL1+/OLIG2+ OPCs with EGFR downregulation (Fig. 6D). These findings, consistent with scRNA-seq analysis, demonstrate that ferret cortical APCs and OPCs share a common Tri-IPC origin (Fig. 6E).

**Figure 7.**
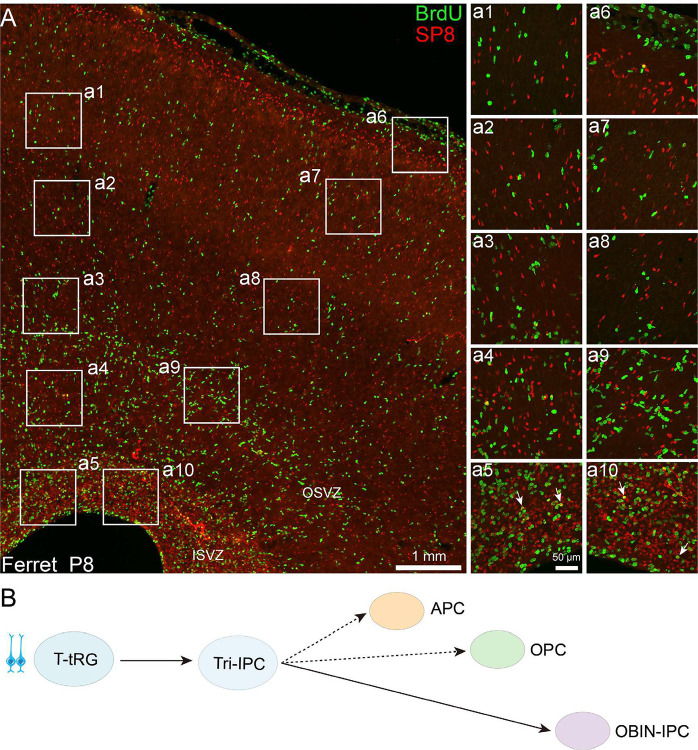
OBIN production in postnatal ferret cortex. **(A)** BrdU/SP8 co-staining 24 hours post-BrdU injection (P7→P8) identifies newly generated OBINs (BrdU+/SP8+ cells, arrows in a5 and a10) exclusively within the ISVZ. No double-positive cells are detected in other cortical regions. **(B)** Lineage model indicates that Tri-IPCs principally produce OBINs in the P8 ferret cortex.

Analysis of scRNA-Seq data revealed no significant population of OBIN-IPCs in the P3 cortex (Fig. S3). We therefore assessed the generation of OBIN-IPCs in the P8 cortex through BrdU labeling (administered at P7 with a 24-hour exposure window) (Fig. 7A). Across all cortical regions examined, BrdU+/SP8+ cells were exclusively localized to the ISVZ, with none detected in the OSVZ or cortical plate (Fig. 7A). This spatial restriction suggests interneuron production is confined to the ISVZ in P8 ferret cortex. The P8 cortical ISVZ contained a high density of SP8+ cells (Fig. 7A), which represent both caudal ganglionic eminence-derived cortical interneurons, and cortically generated neuroblasts for OBINs. Critically, since cortical interneurons cease proliferation upon migration, the BrdU+/SP8+ population in the ISVZ represent newly generated OBINs. These are presumably derived from Tri-IPCs (Fig. 7B), a mechanism conserved in mouse cortex and suggested by human cortical studies^18,19,21^. Collectively, these findings provide evidence that cortical T-tRGs give rise to Tri-IPCs, which serve as multipotent progenitors for APCs, OPCs, and OBIN-IPCs, suggesting an evolutionarily conserved gliogenic and OBIN neurogenesis pathway in mammals.

## DISCUSSION

**Figure 8.**
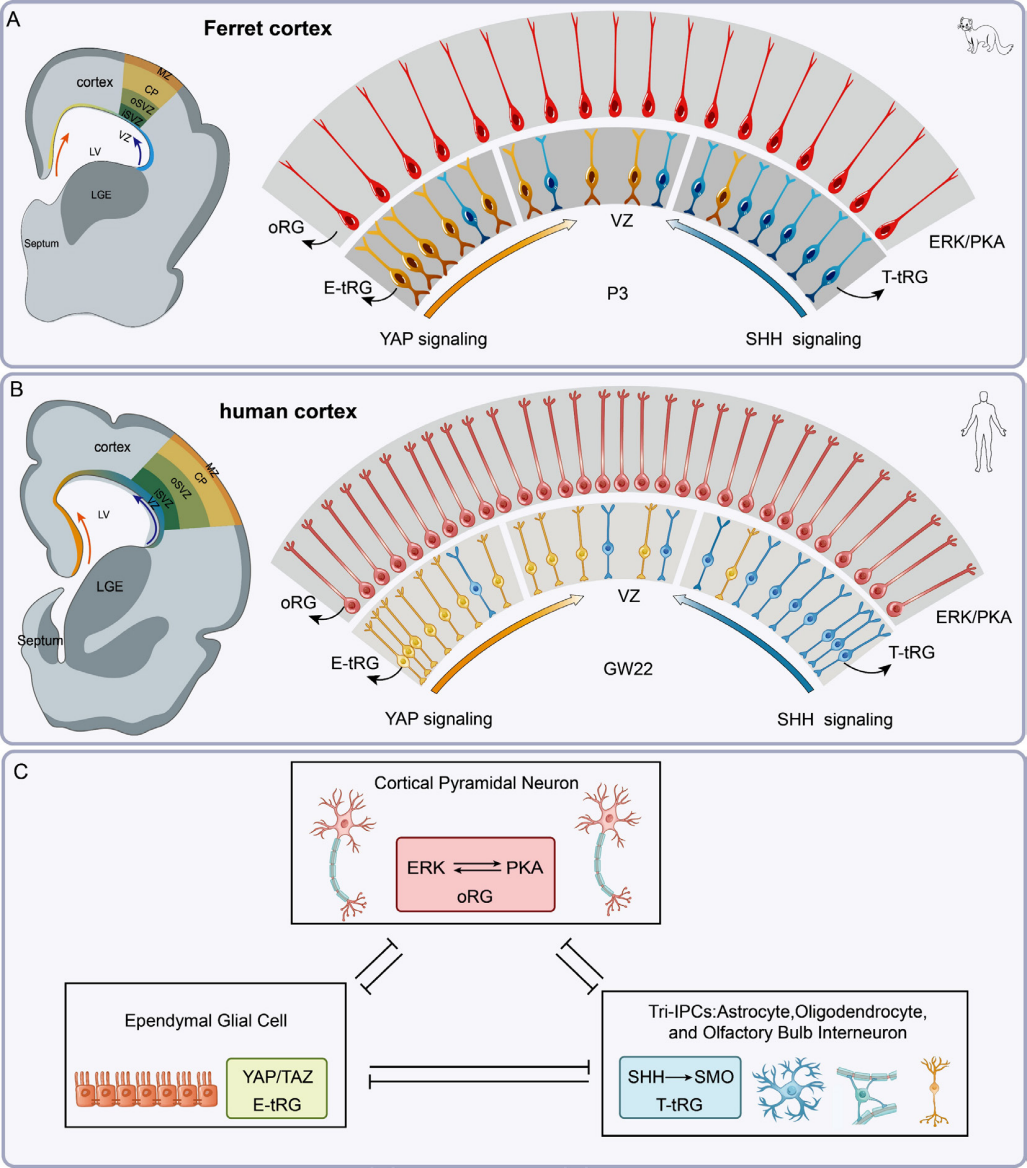
ERK, PKA, YAP, and SHH signaling drives mammalian cortical lineage diversification. **(A, B)** Schematic of cortical RG subtypes in P3 ferret and GW22 human cortex, primarily consisting of oRGs, E-tRGs, and T-tRGs. (**C**) During corticogenesis, ERK/PKA activation in oRGs promotes sustained neurogenesis while suppressing gliogenesis; YAP/TAZ upregulation in E-tRGs drives ependymal cell production; SHH signaling in T-tRGs generates Tri-IPCs that differentiate into astrocytes, oligodendrocytes, and olfactory bulb interneurons. Notably, cortical evolution establishes ERK/PKA as the dominant oRG pathway through a self-reinforcing loop that actively inhibits both YAP and SHH signaling. This evolutionary adaptation provides two key advantages: (1) enhanced oRG self-renewal and (2) prolonged oRG neurogenesis, which drive the expanded neuronal output and neocortical volume, characteristic of human brain evolution. This demonstrates that cortical neurogenesis, gliogenesis, and evolutionary expansion constitute a unified biological program coordinated by ERK/PKA/YAP/SHH signaling.

In this study, we utilize the gyrencephalic ferret model combined with scRNA-Seq analysis of both ferret and human cortices. Through an integrative approach incorporating scRNA-Seq, BrdU labeling, and immunohistochemical analysis, we present three key findings (Fig. 8): (1) Ferret cortical oRGs exhibit enhanced ERK and PKA signaling pathways, predominantly generating cortical PyNs without entering gliogenesis. (2) YAP signaling (YAP/TAZ activity) drives the formation of E-tRGs, with ependymal cell differentiation initiating first in the medial cortex. (3) T-tRGs initiate Tri-IPC production in the lateral cortical VZ. These Tri-IPCs serve as a common progenitor pool for APCs, OPCs, and OBIN-IPCs.

### Molecular characterization and biological implications of oRGs in mammalian cortical development and evolution

Most mammals (except mice and rats) possess a gyrencephalic cortex, which correlates with the presence of oRGs in the OSVZ during cortical development and evolution^4,6,21,26,34,76–79^. oRGs are recognized as fundamental drivers of mammalian cortical expansion. The advent of scRNA-Seq has provided a powerful tool for characterizing the molecular signatures of cortical oRGs, particularly during peak developmental stages in the human brain. Indeed, our recent in-depth analyses have elucidated the fundamental characteristics of oRGs, yielding paradigm-shifting understanding of their developmental roles^15^. Human cortical oRGs demonstrate two distinctive biological properties, exceptional self-renewal potential and an unusually protracted neurogenic window, both of which stem from their characteristically heightened ERK and PKA signaling pathways^15^.

Genetic manipulation in mice demonstrates that ERK signaling deficiency leads to marked depletion of cortical RG populations, culminating in severe microcephaly characterized by dramatically reduced brain volume^15,18,20,80–82^. Moreover, ERK deficiency induces PKA downregulation, releasing its inhibitory effect on YAP signaling. YAP hyperactivation drives precocious conversion of cortical N-RGs into E-RGs, triggering aberrant ependymal differentiation while concurrently depleting both T-tRGs and Tri-IPCs^15,18^. On the other hand, blocking PKA signaling in the mouse cortex induces dramatic upregulation of GLI2/3 transcriptional activators. This molecular switch promotes rapid conversion of cortical N-RGs into T-tRGs, which promptly initiate production of Tri-IPCs that differentiate into APCs, OPCs, and abundant OBIN-IPCs^15,16,18,29^. Thus, the emergence of cortical oRGs in gyrencephalic species, such as ferrets, monkeys, and humans, constitutes an evolutionary adaptation primarily enhancing PyN production, rather than promoting gliogenesis or interneuron generation.

### Cortical RGs exhibit dynamic, spatially varying signaling gradients during development

During cortical development, oRGs in the OSVZ exhibit heightened ERK and PKA pathway activity, in contrast to VZ RGs, where signaling gradients vary spatiotemporally. The medial-high to lateral-low gradient of CRYAB and FOXJ1 expression in ferret cortical VZ E-tRGs serves as a compelling example. Spatiotemporal signaling gradients are essential for orchestrating neocortical development, ensuring controlled expansion and balanced cell-type production. What molecular and cellular mechanisms underlie the formation of these signaling gradients during cortical development?

During cortical patterning, FGF/ERK signaling is activated medially in the rostral cortex, whereas WNT/BMP signaling predominates in the cortical hem, collectively establishing a medial-high to lateral-low signaling gradient^83^. WNT signaling induces *GLI3* expression^84,85^, while ERK signaling potentiates the cAMP-PKA pathway^47–49^. BMP/SMAD signaling also enhances cAMP-PKA pathway^29,86^. FGFR3 and NR2F1 expression display complementary caudal-lateral high to rostral-medial low gradients^83^. Thus, at neurogenesis onset, medial RGs maintain self-renewal through symmetric division, whereas lateral RGs transition to asymmetric division and neurogenesis, a patterning driven by medial-enriched FGF/ERK, PKA, WNT/BMP signaling and GLI3 repressor activity. However, WNT signaling additionally activates YAP signaling through the non-canonical WNT pathway^87^. YAP/TAZ signaling is also progressively amplified by mechanical cues, reduced cell density, and GPCR-mediated inputs (e.g., via GNA12, GNA13 and GNAQ, or GNA11)^55–57^. By the neurogenesis-to-gliogenesis transition stage, these signaling pathways culminate in elevated YAP activity within medial cortical RGs, while lateral cortical RGs exhibit comparatively stronger ERK/PKA signaling. During later cortical development, ventral-derived SHH signaling intensifies in lateral cortical RGs, antagonizing PKA activity and driving the generation of Tri-IPCs.

### YAP signaling drives E-tRG specification and subsequent ependymal lineage progression

Loss of YAP signaling disrupts mouse ependymal cell formation^15,60,61^. On the other hand, juvenile mouse SVZ cells expressing the oncogenic YAP1-MAMLD1 (YAP-M) fusion protein exhibit marked upregulation of YAP pathway components^65^, where YAP/TEAD directly activates the ependymal genetic program^15^. Furthermore, YAP-M strongly suppresses ERK, PKA, and SHH signaling^15,65,88^. These findings suggest that YAP orchestrates a developmental cascade: cortical N-RGs in the medial cortex first transition to E-RGs and then give rise to ependymal cells. In other words, the robust YAP activity in E-RGs imposes a lineage restriction, permitting only ependymal differentiation while blocking competence for T-RG, Tri-IPC, APC, OPC, and OBIN-IPC fates^15^.

### T-RG-derived Tri-IPCs constitute an evolutionarily conserved progenitors for mammalian cortical gliogenesis

SHH-SMO signaling drives the conversion of lateral cortical N-RGs into T-RGs that produce Tri-IPCs. Mouse genetic evidence supports this mechanism: conditional deletion of *Smo* in the mouse cortex markedly reduces production of Tri-IPCs, APCs, OPCs, and OBIN-IPCs, while concomitantly elevating ERK and PKA signaling^15,16,18,20,29,66^. When ERK signaling is inhibited in the mouse cortex, the consequent reduction in PKA activity leads to premature YAP activation in cortical RGs. This early YAP activation precedes ventral-derived SHH signaling and directs cortical N-RGs to adopt an E-RG fate, promoting ependymal differentiation while suppressing T-RG formation^15^. Notably, experimental augmentation of SHH signaling in ERK-deficient cortical RGs rescues T-RG generation and subsequent Tri-IPC production^18^. Thus, SHH-SMO signaling maintains T-RG identity and drives Tri-IPC production through ERK/PKA/YAP inhibition, a mechanism evolutionarily conserved from mice, ferrets, to humans.

In summary, this study suggests that the specification and lineage progression of distinct mammalian cortical RG subtypes constitute a coordinated biological process regulated by an integrated signaling network comprising ERK, PKA, YAP, and SHH pathways (Fig. 8). These evolutionarily conserved signaling cascades collectively drive cortical development and expansion, with ERK as the dominant regulator of this evolutionarily significant developmental program^15^.

## MATERIALS AND METHODS

### Animals

All procedures involving animals were approved by and performed in accordance with the guidelines of the Fudan University Shanghai Medical College Animal Ethics Committee (No. 20230301–141), Shanghai University of Medicine and Health Sciences, and the Institutional Animal Care and Use Committee at University of California at Santa Cruz. Ferrets were purchased from Wuxi Sangosho Biotechnology Co., Ltd. The E33 ferrets were kindly provided by Professor Yong-Chun Yu. Mice were purchased from the Jackson Laboratory or Shanghai Bikeway Biotechnology Co., Ltd. We used *Eomes-CreER*^89^ and *Ai65*^72^ for genetic fate mapping. Ferrets and mice were housed in a 12 h light/12 h dark cycle. The embryonic day of conception was designated as E0.5, with the day of birth defined as postnatal day 0 (P0). Notably, P0 corresponds to E19 in mice and E42 in ferrets at birth.

### Tamoxifen labeling

A single dose of tamoxifen (100 mg/kg) was administered via intraperitoneal injection at postnatal day 0 or 1 (P0/P1), with subsequent analysis performed 20 days post-injection.

### BrdU injection

P7 ferrets received an intraperitoneal injection of BrdU (100 mg/kg) and were perfused 24 hours post-injection at P8 for immunohistochemical analysis.

### In utero electroporation (IUE)

To specifically label dorsal cortical RG-derived Eomes+ cells, we performed in utero electroporation (IUE) at embryonic day 17.0 (E17.0) or postnatal day 0 (P0) to introduce pCAG-Plpo plasmids into the cortical VZ of *Eomes-CreER; Ai65* embryos. CreER activity was induced by subsequent intraperitoneal (i.p.) administration of tamoxifen at P0 or P1, respectively. The pCAG-Flpo plasmid solution (containing 0.05% Fast Green [Sigma-Aldrich] for visualization) was microinjected into the lateral ventricle (0.5 μl per embryo) using a bevelled glass micropipette. Electroporation was conducted using a BTX ECM830 electroporator connected to 5 mm platinum tweezer electrodes (BTX Tweezertrode 45–0488, Harvard Apparatus), applying five square-wave pulses (50 ms duration, 950 ms interval) at 38 V.

### scRNA-Seq library preparation

Following deep anesthesia, cerebral tissues from two P3 ferret pups were rapidly excised and chilled in ice-cold HBSS (Gibco 14175–095). Dorsal cortical regions were isolated, mechanically dissociated into small fragments (<1 mm^3^), and processed to single-cell suspensions via enzymatic digestion (Papain Cell Dissociation Kit) following the standardized protocol, following the manufacturer’s instructions (Miltenyi Biotec, catalog no. 130-092-628). Single-cell RNA sequencing (scRNA-Seq) libraries were prepared using the Chromium droplet-based sequencing platform (10X Genomics), in accordance with the manufacturer’s guidelines (manual document part number: CG00052 Rev C). The cDNA libraries were purified, quantified using an Agilent 2100 Bioanalyzer, and sequenced on an Illumina HiSeq 4000. High-quality sequences (clean reads) were processed using Cell Ranger to obtain quantitative gene expression data. Cellular quality control thresholds were set at 750–5000 genes and <10% mitochondrial transcripts per cell. After filtering, the dataset contained 14,787 cells from two P3 ferret cortices. In parallel, the GW22 human cortex scRNA-Seq dataset was also processed. Raw read counts were normalized using the global scale normalization method, Log Normalize. The normalized datasets were integrated using the Find Integration Anchor and Integration Data functions. Our single-cell RNA sequencing of ferret cortex captured 14,787 high-quality cells (median: 2,000 genes/cell), while analysis of GW22 human cortex yielded 12,567 cells (median: 2,410 genes/cell)^39^. Statistically significant principal components, identified through resampling tests, were retained for Unified Manifold Approximation and Projection (UMAP) analysis. Differentially expressed genes across clusters were identified using the Wilcoxon rank-sum test (adjusted p-value <0.05, |log2FC|>0.25). Cluster annotations were assigned by cross-referencing the most comprehensive and reliable cell type markers from an extensive literature review. All analyses were performed using Seurat v4.0.6. The scRNA-seq data for P3 ferret cortex are available in GEO (GSE304846).

### Tissue preparation

Ferrets and mice were deeply anesthetized and perfused with 4% paraformaldehyde (PFA) in PBS. Embryos were collected from deeply anesthetized pregnant ferrets and mice. All brains were fixed overnight in 4% PFA at 4°C, followed by cryoprotection in 30% sucrose for at least 24 hours. Tissue samples were embedded in Optimal Cutting Temperature (O.C.T.) compound (Sakura Finetek), flash-frozen in a dry ice/ethanol slurry, and stored at −80°C until sectioning. Using a cryostat, we prepared 30 μm sections from all ferret tissues (excluding E33 specimens), while E33 ferret and mouse cortical tissues were sectioned at 20 μm.

High-quality human cortical sections (GW19: n=1; GW23: n=2; 60 μm) were employed for immunostaining in this study. The GW19 specimen, initially classified as GW18, was re-designated following comprehensive immunohistochemical re-evaluation. All sections have been fully characterized in our earlier studies^18,20,21,29,90,91^. All sections were mounted on glass slides for staining.

### Immunofluorescence

The tissue sections were first washed with 0.05 M TBS for 10 minutes, then incubated in Triton-X-100 (0.5% in 0.05 M TBS) for 30 minutes at room temperature (RT). Following this, the sections were incubated with blocking solution (5% donkey serum + 0.5% Triton-X-100 in 0.05 M TBS, pH 7.2) for 2 hours at RT. Primary antibodies, diluted in 5% donkey serum, were applied and incubated overnight at 4°C. Afterward, the sections were rinsed three times with 0.05 M TBS. Secondary antibodies (1:500, all from Jackson ImmunoResearch) specific to the appropriate species were added and incubated for 2 hours at RT. Fluorescently stained sections were then washed three times with 0.05 M TBS for 10 minutes each. The sections were subsequently stained with 4’,6-diamidino-2-phenylindole (DAPI) (1:3000, Sigma) diluted in TBS for 1 minute and rinsed three more times with TBS. Finally, the sections were coverslipped with Gel/Mount (Biomeda, Foster City, CA).

The following primary antibodies were used in this study: rat Anti-BrdU (1:200, Accurate Chemical and Scientific Corporation, OBT0030S), rabbit anti-ASCL1 (1:1000, Cosmo Bio, SKT01–003), guinea pig anti-DLX2 (1:250, Oasis Biofarm, OB-PGP017), goat anti-EGFR (1:1000, R&D System, BAF1280), goat anti tdT (tdTomato, 1:2000, SICGEN, Ab8181), rabbit anti-OLIG2 (1:500, Millipore, AB9610), rat anti-OLIG2 (1:500, Oasis Biofarm, OB-PRB009–01), rabbit anti-pERK1/2 (pERK, 1:400, Cell Signaling Technology, Cat# 4370), mouse anti-PAX6 (1:1000, BD Pharmingen^™^, Cat# 561664), guinea pig anti-EOMES (TBR2, 1:500, Oasis Biofarm, OB-PGP022), rat anti-EOMES (1:500, Thermo Fisher, Cat# 12-4875-82), goat anti-SP8 (1:3000, Santa Cruz, Sc-104661), mouse anti CRYAB (1:300, Abcam, ab13496); rabbit anti-HOPX (1:500, Proteintech, Cat# 11419–1-AP), rat anti-HOPX (1:500, Oasis Biofarm, OB-PRT015), and mouse anti FOXJ1 (1:1000, Invitrogen Cat# 14-9965-80).

### Image acquisition

Whole-slide imaging was performed using an Olympus VS120 slide scanner with 10× or 20× objectives. High-resolution fluorescence imaging was conducted on an Olympus FV3000 confocal microscope system equipped with 10×, 20×, and 40× objectives. All images were processed (merged, cropped, and contrast-adjusted) using Adobe Photoshop and Illustrator software while maintaining data integrity.

## Supplementary Material

Additional information

Supporting Information is available online or from the author.

Supplementary Files

This is a list of supplementary files associated with this preprint. Click to download.

- FerretSupplementaryMaterials.pdf
- Tables.zip

## Data Availability

The scRNA-Seq data from P3 ferret cortex were generated during the current study are available in the Gene Expression Omnibus (GEO: GSE304846). The scRNA-Seq data from GW22 human cortex (GSE162170) were previously published^39^. All remaining data are presented either in the main manuscript or supplementary materials.
